# Supplementary material for: Theoretical studies on quantum imaging with time-integrated single-photon detection under realistic experimental conditions
Source: Sci Rep. 2022 Mar 29;12:5338. doi: 10.1038/s41598-022-09186-w (PMC8964742; doi:10.1038/s41598-022-09186-w)
Supplement: Supplementary file 1 — Supplementary Information. [file 41598_2022_9186_MOESM1_ESM.pdf]

Theoretical studies on quantum imaging with time-integrated single-photon detection under realistic experimental conditions - Supplementary

Byeong-Yoon Go[1,*], Changhyoup Lee[2,†], and Kwang-Geol Lee[1,‡]

[1]Department of Physics, Hanyang University, Seoul 04763, Republic of Korea
[2]Korea Research Institute of Standards and Science, Daejeon 34113, Republic of Korea
[*]Present address: Korea Advanced Institute of Science and Technology, Daejeon 34141, Republic of Korea
[†]changhyoup.lee@gmail.com
[‡]kglee@hanyang.ac.kr

In this supplementary material, we show that the SNR* of Eq. (2) calculated for PNRD is approximately equal to the SNR* obtainable by the SPCM.

SNR* for click statistics

When two local SPCMs are performed m times onto a two-mode state ρ, they produce two sequential outcomes with a length of m for the individual modes, and the outcomes are recorded in terms of 0 (i.e., 'no click') and 1 (i.e., 'click'). The number of click events is then counted in each mode, so that we consider two-mode click statistics $\{c_1, c_2\}$ for the numbers of click events $c_j \in \{0, 1, \cdots m\}$. The statistical features of the click statistics need to be implemented in the calculation of SNR and SNR* for a given state ρ.

To this end, let us first consider the probability of the counts $\nu = (\nu_{00}, \nu_{01}, \nu_{10}, \nu_{11})$ for the set of individual detection outcomes $\{00, 01, 10, 11\}$ out of m sequential measurements. The subscript denotes the "no click" event for 0 and "click" event for 1, each for signal channel at the first place, and reference channel at the second place of the subscript. It follows the multinomial distribution written as

$$P(\nu) = \frac{m!}{\nu_{00}!\nu_{01}!\nu_{10}!\nu_{11}!} P_{00}{}^{\nu_{00}} P_{01}{}^{\nu_{01}} P_{10}{}^{\nu_{10}} P_{11}{}^{\nu_{11}},$$

where $\|\nu\|_1 = m,\ P_\mu = \mathrm{Tr}[\hat{\pi}_\mu \rho]$ represents the probability for the outcome μ at individual measurements, and $\hat{\pi}_\mu = |\mu\rangle\langle\mu|$ denotes the positive operator-valued measure (POVM) projector corresponding to the outcome $\mu \in \{00, 01, 10, 11\}$. Then, the expectation value, variance, and covariance for the counts of individual events are written as

$$\begin{aligned} \mathrm{E}(\nu_\mu) &= mP_\mu, \\ \mathrm{Var}(\nu_\mu) &= mP_\mu(1 - P_\mu), \\ \mathrm{Cov}(\nu_\mu, \nu_\beta) &= -mP_\mu P_\beta, \end{aligned}$$

respectively. Therefore, the expectation value and the variance of the click counts c_j for each mode can be written as

$$\begin{aligned} \langle c_1 \rangle &= \mathrm{E}(\nu_{10}) + \mathrm{E}(\nu_{11}) = m(P_{10} + P_{11}), \\ \langle c_2 \rangle &= \mathrm{E}(\nu_{01}) + \mathrm{E}(\nu_{11}) = m(P_{01} + P_{11}), \\ \langle \Delta^2 c_1 \rangle &= \mathrm{Var}(\nu_{10}) + \mathrm{Var}(\nu_{11}) + 2\mathrm{Cov}(\nu_{10}, \nu_{11}) = m\left[P_{10} + P_{11} - (P_{10} + P_{11})^2\right], \\ \langle \Delta^2 c_2 \rangle &= \mathrm{Var}(\nu_{01}) + \mathrm{Var}(\nu_{11}) + 2\mathrm{Cov}(\nu_{01}, \nu_{11}) = m\left[P_{01} + P_{11} - (P_{01} + P_{11})^2\right]. \end{aligned}$$

Here, it is obvious that the click statistics can be calculated for any state ρ, but is different from the true photon number statistics that is obtainable by PNRD. For the differential imaging scheme, the expectation value and the variance of the differential click

numbers between c_1 and c_2 are then given as

$$I_- = \langle c_- \rangle = \mathrm{E}(\nu_{01}) - \mathrm{E}(\nu_{10}) = m(P_{01} - P_{10}),$$
$$\Gamma_- = I_-(\alpha + \delta\alpha) - I_-(\alpha) = m(\delta P_{01} - \delta P_{10}),$$
$$(\Delta I_-)^2 = \langle (\Delta c_-)^2 \rangle = \mathrm{Var}(\nu_{01}) + \mathrm{Var}(\nu_{10}) - 2\mathrm{Cov}(\nu_{10}, \nu_{01}) = m\left[P_{10} + P_{01} - (P_{10} - P_{01})^2\right],$$

where $\delta P_\mu = P_\mu(\alpha + \delta\alpha) - P_\mu(\alpha)$ and $P_\mu(\alpha) = \mathrm{Tr}[\hat{\pi}_\mu \rho(\alpha)]$ for a state ρ having undergone absorption with α. The SNR* is then

$$\mathrm{SNR}^* = \frac{\Gamma_-}{\Delta I_-} = \frac{\sqrt{m}(\delta P_{01} - \delta P_{10})}{\sqrt{P_{10} + P_{01} - (P_{10} - P_{01})^2}}. \tag{A1}$$

The calculation of SNR* is now straightforward for any state ρ. Normally the effect of detection inefficiency and dark counts are included in the POVM elements via proper modification of a detector model, but here we include them into a quantum state ρ for convenience, as further discussed below.

Lossy and noisy quantum states with $N \ll 1$

For the calculation of SNR* of Eq. (A1), here we include the effect of loss, absorption, and dark count into a two-mode quantum state ρ. Let us begin with assuming equal loss rates $\gamma_1 = \gamma_2 = \gamma$, equal mean dark counts $\langle \hat{n}_{d1} \rangle = \langle \hat{n}_{d2} \rangle = N_d$, and a twin-mode input state, i.e., $\langle \hat{n}_1 \rangle = \langle \hat{n}_2 \rangle = N$, as in the main text for simplicity. We further assume that the contribution from higher photon numbers than a single photon is negligible, i.e., $N \ll 1$. In the limit of small N, we can write an arbitrary twin-mode quantum state as

$$|\Psi\rangle \approx \sum_{j,k=0}^{1} c_{j,k} |j,k\rangle, \tag{B1}$$

for which $\sum_{j,k=0}^{1} |c_{j,k}|^2 \approx 1$ and $\langle \Psi | \hat{n}_{1,2} | \Psi \rangle \approx N \ll 1$. Since SPCM (and even PNRD) access only the diagonal elements of a density matrix $|\Psi\rangle\langle\Psi|$, an effective density matrix for the state of Eq. (B1) can be written in an operational sense as

$$\rho_{\mathrm{in}} \simeq p_{00}\hat{\pi}_{00} + p_{01}\hat{\pi}_{01} + p_{10}\hat{\pi}_{10} + p_{11}\hat{\pi}_{11}, \tag{B2}$$

where $p_{jk} = |c_{jk}|^2$ denotes the population of the state $|j,k\rangle$ in ρ and here the projectors $\hat{\pi}_{jk}$ are used as the photon-number basis of a density matrix. Here, p_{jk} are determined for a given input state.

The absorption (α) and loss (γ) can be implemented by modifying $p_{jk} \to p_{jk}(\alpha, \gamma)$ in the ideal state of Eq. (B2), where

$$p_{00}(\alpha,\gamma) = p_{00} + \gamma p_{01} + [\alpha + \gamma(1-\alpha)]p_{10} + \gamma[\alpha + \gamma(1-\alpha)]p_{11},$$
$$p_{01}(\alpha,\gamma) = (1-\gamma)(p_{01} + [\alpha + \gamma(1-\alpha)]p_{11}),$$
$$p_{10}(\alpha,\gamma) = (1-\alpha)(1-\gamma)(p_{10} + \gamma p_{11}),$$
$$p_{11}(\alpha,\gamma) = (1-\alpha)(1-\gamma)^2 p_{11},$$

leading to

$$\rho_{\mathrm{lossy}} \simeq p_{00}(\alpha,\gamma)\hat{\pi}_{00} + p_{01}(\alpha,\gamma)\hat{\pi}_{01} + p_{10}(\alpha,\gamma)\hat{\pi}_{10} + p_{11}(\alpha,\gamma)\hat{\pi}_{11}. \tag{B3}$$

The dark count can be modeled by an inflow of a weak coherent state in addition to the actual states being measured, so it can be implemented by modifying $\hat{\pi}_{jk} \to \hat{\pi}'_{jk}$ in the lossy state of Eq. (B3), where

$$\hat{\pi}'_{00} = e^{-2N_d}\hat{\pi}_{00} + e^{-N_d}(1 - e^{-N_d})(\hat{\pi}_{01} + \hat{\pi}_{10}) + (1 - e^{-N_d})^2\hat{\pi}_{11},$$
$$\hat{\pi}'_{01} = e^{-N_d}\hat{\pi}_{01} + (1 - e^{-N_d})\hat{\pi}_{11},$$
$$\hat{\pi}'_{10} = e^{-N_d}\hat{\pi}_{10} + (1 - e^{-N_d})\hat{\pi}_{11},$$
$$\hat{\pi}'_{11} = \hat{\pi}_{11}.$$

Here, N_d is the average inflow photon number. Therefore, a lossy and noisy state through the modification introduced above can be written as

$$\rho_{\mathrm{lossy\&noisy}} \simeq p_{00}(\alpha,\gamma)\hat{\pi}'_{00} + p_{01}(\alpha,\gamma)\hat{\pi}'_{01} + p_{10}(\alpha,\gamma)\hat{\pi}'_{10} + p_{11}(\alpha,\gamma)\hat{\pi}'_{11}. \tag{B4}$$

Investigation of various input states

The state of Eq. (B4) is of the general form for an arbitrary two-mode state when $N \ll 1$. Here we investigate the SNR*'s of Eq. (A1) for various input states and compare those with SNR* for the true photon number statistics. For the calculation of SNR*'s with respect to a state ρ of Eq. (B4), one can write $P_\mu = \mathrm{Tr}[\hat{\pi}_\mu \rho]$ for $\mu \in \{00, 01, 10, 11\}$:

$$P_{00}(\alpha,\gamma) = e^{-2N_{\rm d}} p_{00}(\alpha,\gamma), \tag{C1}$$
$$P_{01}(\alpha,\gamma) = e^{-N_{\rm d}} p_{01}(\alpha,\gamma) + e^{-N_{\rm d}}(1-e^{-N_{\rm d}}) p_{00}(\alpha,\gamma), \tag{C2}$$
$$P_{10}(\alpha,\gamma) = e^{-N_{\rm d}} p_{10}(\alpha,\gamma) + e^{-N_{\rm d}}(1-e^{-N_{\rm d}}) p_{00}(\alpha,\gamma), \tag{C3}$$
$$P_{11}(\alpha,\gamma) = p_{11}(\alpha,\gamma) + (1-e^{-N_{\rm d}})\left[p_{01}(\alpha,\gamma) + p_{10}(\alpha,\gamma)\right] + (1-e^{-N_{\rm d}})^2 p_{00}(\alpha,\gamma), \tag{C4}$$

where $p_\mu(\alpha,\gamma)$ are to be set by a given input state. Below, writing $p_\mu(\alpha,\gamma)$ for the three states considered in the main text, we obtain $P_\mu(\alpha,\gamma)$ using Eqs. (C1)-(C4), leading to the calculation of SNR*'s.

Coherent state input

The coherent state input can be written in the form of Eq. (B4) as $\rho^{\rm coh} \simeq e^{-2N}\hat{\pi}_{00} + e^{-N}(1-e^{-N})(\hat{\pi}_{01}+\hat{\pi}_{10}) + (1-e^{-N})^2\hat{\pi}_{11}$, i.e.,

$$\begin{aligned}
p_{00} &= e^{-2N},\\
p_{01} &= p_{10} = e^{-N}(1-e^{-N}),\\
p_{11} &= (1-e^{-N})^2,
\end{aligned}$$

leading to

$$\begin{aligned}
p_{00}(\alpha,\gamma) &= e^{-2N} + [\alpha+\gamma(2-\alpha)]e^{-N}(1-e^{-N}) + \gamma(\alpha+\gamma-\alpha\gamma)(1-e^{-N})^2,\\
p_{01}(\alpha,\gamma) &= (1-\gamma)(1-e^{-N})[e^{-N} + (\alpha+\gamma-\alpha\gamma)(1-e^{-N})],\\
p_{10}(\alpha,\gamma) &= (1-\alpha)(1-\gamma)(1-e^{-N})[e^{-N} + \gamma(1-e^{-N})],\\
p_{11}(\alpha,\gamma) &= (1-\alpha)(1-\gamma)^2(1-e^{-N})^2.
\end{aligned}$$

Therefore, P_μ's are written as

$$\begin{aligned}
P_{00}(\alpha,\gamma) &= e^{-2N_{\rm d}}\{e^{-N} + \gamma(1-e^{-N})\}\left[e^{-N} + (\alpha+\gamma-\alpha\gamma)(1-e^{-N})\right],\\
P_{01}(\alpha,\gamma) &= e^{-N_{\rm d}}\{1 - e^{-N-N_{\rm d}} - \gamma e^{-N_{\rm d}}(1-e^{-N})\}\left[e^{-N} + (\alpha+\gamma-\alpha\gamma)(1-e^{-N})\right],\\
P_{10}(\alpha,\gamma) &= e^{-N_{\rm d}}\{e^{-N} + \gamma(1-e^{-N})\}\left[1 - (\alpha+\gamma-\alpha\gamma)e^{-N_{\rm d}} - (1-\alpha)(1-\gamma)e^{-N-N_{\rm d}}\right],\\
P_{11}(\alpha,\gamma) &= \{1 - e^{-N-N_{\rm d}} - \gamma e^{-N_{\rm d}}(1-e^{-N})\}\left[1 - (\alpha+\gamma-\alpha\gamma)e^{-N_{\rm d}} - (1-\alpha)(1-\gamma)e^{-N-N_{\rm d}}\right].
\end{aligned}$$

The signal and variance for coherent state are then

$$\begin{aligned}
\Gamma_-^{\rm coh} &= m\delta\alpha e^{-N_{\rm d}}(1-\gamma)(1-e^{-N}),\\
(\Delta I_-^{\rm coh})^2 &= me^{-N_{\rm d}}[\alpha(1-\gamma) + 2\gamma + e^{-N}(2-\alpha)(1-\gamma) - e^{-2N-N_{\rm d}}(2-\alpha(2-\alpha))(1-\gamma)^2\\
&\quad - 2e^{-N-N_{\rm d}}(1-\gamma)(\alpha-\alpha^2(1-\gamma)+2\gamma-2\alpha\gamma) - e^{-N_{\rm d}}(\alpha^2(1-\gamma)^2 + 2\alpha\gamma(1-\gamma) + 2\gamma^2)].
\end{aligned}$$

TMSV state input

For a TMSV state, the photon numbers are completely correlated between the two modes, so it can be written in the form of Eq. (B4) as

$$\rho^{\rm TMSV} \simeq p_{00}\hat{\pi}_{00} + p_{11}\hat{\pi}_{11},$$

where $p_{00} = e^{-N}$ [i.e., $p_{01} = p_{10} = 0$ and $p_{11} = (1-e^{-N})$], originated from the Poisson distribution due to the multi-mode feature of a TMSV state. This leads to

$$\begin{aligned}
p_{00}(\alpha,\gamma) &= e^{-N} + \gamma(\alpha+\gamma-\alpha\gamma)(1-e^{-N}),\\
p_{01}(\alpha,\gamma) &= (1-\gamma)(\alpha+\gamma-\alpha\gamma)(1-e^{-N}),\\
p_{10}(\alpha,\gamma) &= (1-\alpha)\gamma(1-\gamma)(1-e^{-N}),\\
p_{11}(\alpha,\gamma) &= (1-\alpha)(1-\gamma)^2(1-e^{-N}).
\end{aligned}$$

Therefore, P_μ's are written

$$
\begin{aligned}
P_{00}(\alpha,\gamma) &= e^{-2N_\mathrm{d}}\left[e^{-N}+\gamma(\alpha+\gamma-\alpha\gamma)(1-e^{-N})\right],\\
P_{01}(\alpha,\gamma) &= e^{-N_\mathrm{d}}\left[(\alpha+\gamma-\alpha\gamma)(1-\gamma e^{-N_\mathrm{d}})-e^{-N}(1-\gamma)\{e^{-N_\mathrm{d}}-(1-\alpha)(1-\gamma e^{-N_\mathrm{d}})\}\right],\\
P_{10}(\alpha,\gamma) &= e^{-N_\mathrm{d}}\left[\gamma\{1-(\alpha+\gamma-\alpha\gamma)e^{-N_\mathrm{d}}\}+e^{-N}(1-\gamma)\{1-e^{-N_\mathrm{d}}-\gamma(1-\alpha)e^{-N_\mathrm{d}}\}\right],\\
P_{11}(\alpha,\gamma) &= (1-\gamma e^{-N_\mathrm{d}})\{1-(\alpha+\gamma-\alpha\gamma)e^{-N_\mathrm{d}}\}+e^{-N-N_\mathrm{d}}(1-\gamma)\{e^{-N_\mathrm{d}}-(2-\alpha)+\gamma(1-\alpha)e^{-N_\mathrm{d}}\}.
\end{aligned}
$$

The signal and variance for the TMSV state input are then

$$
\begin{aligned}
\Gamma_-^{\mathrm{TMSV}} &= m\delta\alpha e^{-N_\mathrm{d}}(1-\gamma)(1-e^{-N})\\
(\Delta I_-^{\mathrm{TMSV}})^2 &= me^{-N_\mathrm{d}}\left[2e^{-N}(1-e^{-N_\mathrm{d}})+(1-e^{-N})\{\alpha(1-\gamma)+2\gamma-e^{-N_\mathrm{d}}\left(2\gamma(\alpha+\gamma-\alpha\gamma)+\alpha^2(1-e^{-N})(1-\gamma)^2\right)\}\right].
\end{aligned}
$$

TF state input

Although the state of Eq. (B4) can be used for an arbitrary two-mode state, we here consider the TF state input as the case that leads to mN detection events among m time bins with an assumption $mN \in \mathbb{Z}$ and $N \ll 1$. In other words, one can consider that a sequence of mN TF states fires the click events at individual time bins among m bins, but randomly distributed. Such an assumption is set for a reasonable comparison with the other states considered above. At time bins where the TF state is not given, the click event can also be fired due to the dark count contribution. The statistical features of the click number distribution are thus given as

$$
\begin{aligned}
E\left(\nu_\mu\right) &= mNP_\mu+m(1-N)P_\mu^{\mathrm{dark}},\\
\mathrm{Var}\left(\nu_\mu\right) &= mNP_\mu(1-P_\mu)+m(1-N)P_\mu^{\mathrm{dark}}(1-P_\mu^{\mathrm{dark}}),\\
\mathrm{Cov}\left(\nu_\mu,\nu_\beta\right) &= -mNP_\mu P_\beta,
\end{aligned}
$$

where $P_\mu = \mathrm{Tr}(\rho\hat{\pi}_\mu)$ for the TF state ρ and $P_\mu^{\mathrm{dark}} = \mathrm{Tr}(\rho^{\mathrm{dark}}\hat{\pi}_\mu)$ for an effective coherent state ρ^{dark} causing the dark count. Here, the second terms are due to dark count contribution for $m(1-N)$ time bins and there is no correlation between the contributions from ρ and ρ^{dark}.

The SPCM cannot distinguish between a single photon and more photons, the TF state can be considered as $\rho^{\mathrm{TF}} = \hat{\pi}_{11}$, i.e., a pair of single-photon states, for which

$$
\begin{aligned}
p_{00} &= p_{01} = p_{10} = 0,\\
p_{11} &= 1,
\end{aligned}
$$

leading to

$$
\begin{aligned}
p_{00}(\alpha,\gamma) &= \gamma(\alpha+\gamma-\alpha\gamma),\\
p_{01}(\alpha,\gamma) &= (1-\gamma)(\alpha+\gamma-\alpha\gamma),\\
p_{10}(\alpha,\gamma) &= \gamma(1-\alpha)(1-\gamma),\\
p_{11}(\alpha,\gamma) &= (1-\alpha)(1-\gamma)^2.
\end{aligned}
$$

Therefore, P_μ's are written as

$$
\begin{aligned}
P_{00}(\alpha,\gamma) &= e^{-2N_\mathrm{d}}\gamma(\alpha+\gamma-\alpha\gamma),\\
P_{01}(\alpha,\gamma) &= e^{-N_\mathrm{d}}(1-\gamma e^{-N_\mathrm{d}})(\alpha+\gamma-\alpha\gamma),\\
P_{10}(\alpha,\gamma) &= e^{-N_\mathrm{d}}\gamma\left[1-(\alpha+\gamma-\alpha\gamma)e^{-N_\mathrm{d}}\right],\\
P_{11}(\alpha,\gamma) &= (1-\gamma e^{-N_\mathrm{d}})\left[1-(\alpha+\gamma-\alpha\gamma)e^{-N_\mathrm{d}}\right].
\end{aligned}
$$

The signal and variance for the TF state input are then

$$
\begin{aligned}
\Gamma_-^{\mathrm{TF}} &= mN\delta\alpha e^{-N_\mathrm{d}}(1-\gamma),\\
(\Delta I_-^{\mathrm{TF}})^2 &= me^{-N_\mathrm{d}}\left[2(1-e^{-N_\mathrm{d}})-N(2-\alpha)(1-\gamma)-Ne^{-N_\mathrm{d}}(1-\gamma)\{\alpha^2(1-\gamma)+2\alpha\gamma-2\gamma-2\}\right],
\end{aligned}
$$

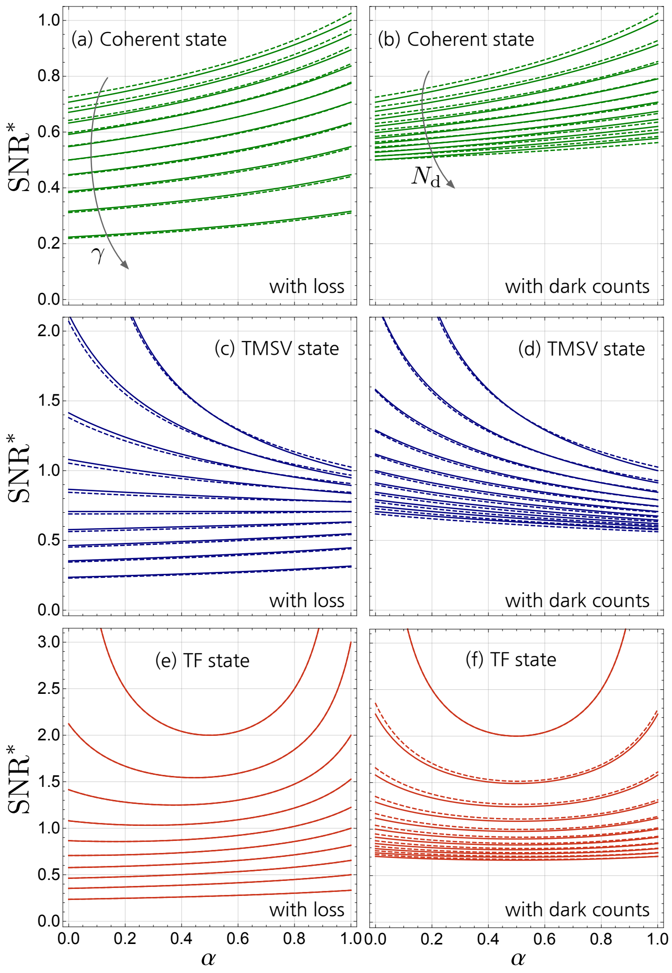

Figure S1. SNR* as a function of α for various input states (respective rows) when using SPCMs (dashed curves) and PNRD (solid curves) with loss (left column) and the dark counts (right column). Here, we consider $\gamma = 0, 0.1, \cdots, 0.9$ for loss and $N_\mathrm{d} = 0, 0.01, \cdots, 0.1$ for the dark counts, showing that SNR* for all the cases generally decreases with loss and dark counts. Particularly for SPCMs, $m = 10^7$ and $\delta\alpha = 10^{-3}$ have used.

SNR*

Figure S1 presents the comparison of SNR* between the two detection schemes: SPCMs of Eq. (A1) and PNRD of Eq. (5) for the three input states (see the respective rows) with loss (see the left panel) and with the dark count (see the right panel). For each input state, the cases using SPCMs and PNRD are represented by dashed and solid curves, respectively. It is clear to see that the two detection schemes are very similar in most cases. This indicates that the detection scheme using SPCMs for a weak input field (i.e., $N \ll 1$) can effectively realize the detection scheme using PNRD, which justifies the use of Eq. (5) for the scheme using SPCMs in good approximation for simplicity and convenience.
